# Effects of Dynamic Neck Sensorimotor Biofeedback Training in Individuals With Mechanical Neck Pain: A Pilot Randomized Controlled Trial

**DOI:** 10.1002/pri.70322

**Published:** 2026-08-18

**Authors:** Min Hsu, Yen‐Cheng Lee, Chia‐Chi Yang, Pei‐Chi Lin, Lih‐Jiun Liaw, Chen‐Yu Chien, Pei‐Yun Lee, Lan‐Yuen Guo

**Affiliations:** ^1^ Department of Sports Medicine College of Medicine Kaohsiung Medical University Kaohsiung Taiwan; ^2^ The Master Program of Long‐Term Care in Aging College of Nursing Kaohsiung Medical University Kaohsiung Taiwan; ^3^ Center for Long‐Term Care Research Kaohsiung Medical University Kaohsiung Taiwan; ^4^ Department of Physical Therapy College of Health Sciences Kaohsiung Medical University Kaohsiung Taiwan; ^5^ Department of Otorhinolaryngology Kaohsiung Medical University Hospital Kaohsiung Medical University Kaohsiung Taiwan; ^6^ Department of Otorhinolaryngology School of Medicine College of Medicine Kaohsiung Medical University Kaohsiung Taiwan; ^7^ Department of Physical Therapy College of Medicine National Cheng Kung University Tainan Taiwan; ^8^ Ph.D. Program in Biomedical Engineering College of Medicine Kaohsiung Medical University Kaohsiung Taiwan; ^9^ Precision Sports Medicine and Health Promotion Center Kaohsiung Medical University Kaohsiung Taiwan; ^10^ College of Health Sciences Kaohsiung Medical University Kaohsiung Taiwan; ^11^ Research Center for Precision Environmental Medicine Kaohsiung Medical University Kaohsiung Taiwan; ^12^ Department of Medical Research Kaohsiung Medical University Hospital Kaohsiung Taiwan; ^13^ College of Humanities and Social Sciences National Pingtung University of Science and Technology Pingtung Taiwan

**Keywords:** biofeedback training, fear‐avoidance beliefs, mechanical neck pain, proprioception training, repositioning error

## Abstract

Mechanical neck pain (MNP) is commonly accompanied by pain‐related functional limitations, sensorimotor disturbances, and fear of movement, which together may contribute to persistent disability. This preliminary randomized controlled trial study investigated the short‐term effects of dynamic neck sensorimotor‐based biofeedback training in individuals with MNP. 20 MNP patients from outpatient clinics were assigned to a biofeedback training group or a control group. The training group underwent dynamic biofeedback exercises twice weekly for 2 weeks, whereas the control group performed repeated cervical movements without biofeedback. Outcomes included cervical kinematics as repositioning errors (RPE), movement units (MU), maximal range of motion (ROM), and subjective measures, including pain intensity, Neck Disability Index (NDI), and Fear‐Avoidance Beliefs Questionnaire (FABQ). All participants completed post‐intervention assessments; adherence in the training group was 100%, with no missing data and no adverse events reported. Within the biofeedback training group, participants receiving biofeedback training demonstrated greater improvements in cervical repositioning accuracy during flexion (51.95%, *p* = 0.04) and extension (46.67%, *p* = 0.02), along with reductions in fear‐avoidance beliefs related to physical activity and work (*p* < 0.05); these changes were less apparent in the active control group. Exploratory regression analyses suggested associations between improvements in repositioning accuracy and pain reduction, and between increased cervical range of motion and improvements in fear‐avoidance beliefs related to physical activity. These pilot findings suggest that dynamic sensorimotor biofeedback training may improve proprioceptive acuity and fear‐avoidance beliefs in individuals with MNP, supporting further evaluation in an adequately powered randomized trial.

## 1 Introduction

Mechanical neck pain (MNP) is one of the most prevalent musculoskeletal disorders, affecting 30%–50% of the global population (Hogg‐Johnson et al. 2008). Beyond pain symptoms, MNP is frequently accompanied by proprioceptive dysfunction, which manifests as increased joint repositioning errors, reduced cervical range of motion (ROM), and impaired postural control (Banks et al. 2021; Sjolander et al. 2008). These deficits are thought to arise from abnormal proprioceptive input that disrupts sensorimotor integration, that is, the process by which sensory input guides motor output for posture and movement control (Peng et al. 2021; Proske and Gandevia 2012). Patients with MNP often exhibit reduced proprioceptive sensitivity and increased irregularity during cervical movements, which further compromise postural stability (Clark et al. 2015; Treleaven et al. 2003). Such dysfunction is thought to be related to persistent neck pain and the risk of chronicity. However, the direction of this relationship remains uncertain. Sensorimotor deficits are frequently observed in MNPs, but existing evidence largely supports an association rather than a confirmed causal pathway (Qu et al. 2022). Accordingly, it remains unclear whether sensorimotor dysfunction is a driver of pain, a consequence of pain‐related protective behavior, or both.

Proprioceptive impairments are closely related to functional limitations in MNPs. Increased cervical repositioning error has been reported in individuals with neck pain and may co‐exist with restricted ROM, impaired movement smoothness, postural instability, and disability‐related outcomes (Cuenca‐Martínez et al. 2018; de Vries et al. 2015; Rahnama et al. 2023). Although previous studies have supported the use of proprioceptive and postural correction training, most have focused on static tasks or relied on a single feedback modality, such as visual cues alone. This represents an important limitation, as effective motor relearning typically requires enriched and task‐specific feedback to facilitate postural correction and sensorimotor adaptation during dynamic movement. In contrast, training programs that incorporate multiple feedback modalities, such as visual and auditory cues, and progressively reduce external feedback may better promote internal proprioceptive control and motor learning (G. Jull et al. 2007; Saadat et al. 2019). However, empirical evidence supporting the effectiveness of such multimodal, progressively structured sensorimotor training in individuals with MNP remains limited (Breen et al. 2009; Shirvani et al. 2021).

To address this gap, the present study investigated the short‐term effects of a sensorimotor training program combining static and dynamic cervical movement tasks with progressively reduced visual and auditory biofeedback. The study aimed to examine whether this training approach would lead to improvements in cervical proprioceptive acuity as well as other kinematic outcomes, including range of motion and movement smoothness, and clinical outcomes, including pain intensity, neck‐related disability, and fear‐avoidance beliefs. Exploratory analyses examined associations between changes in movement‐related measures and patient‐reported outcomes.

## 2 Methods

### 2.1 Study Design

**FIGURE 1:**
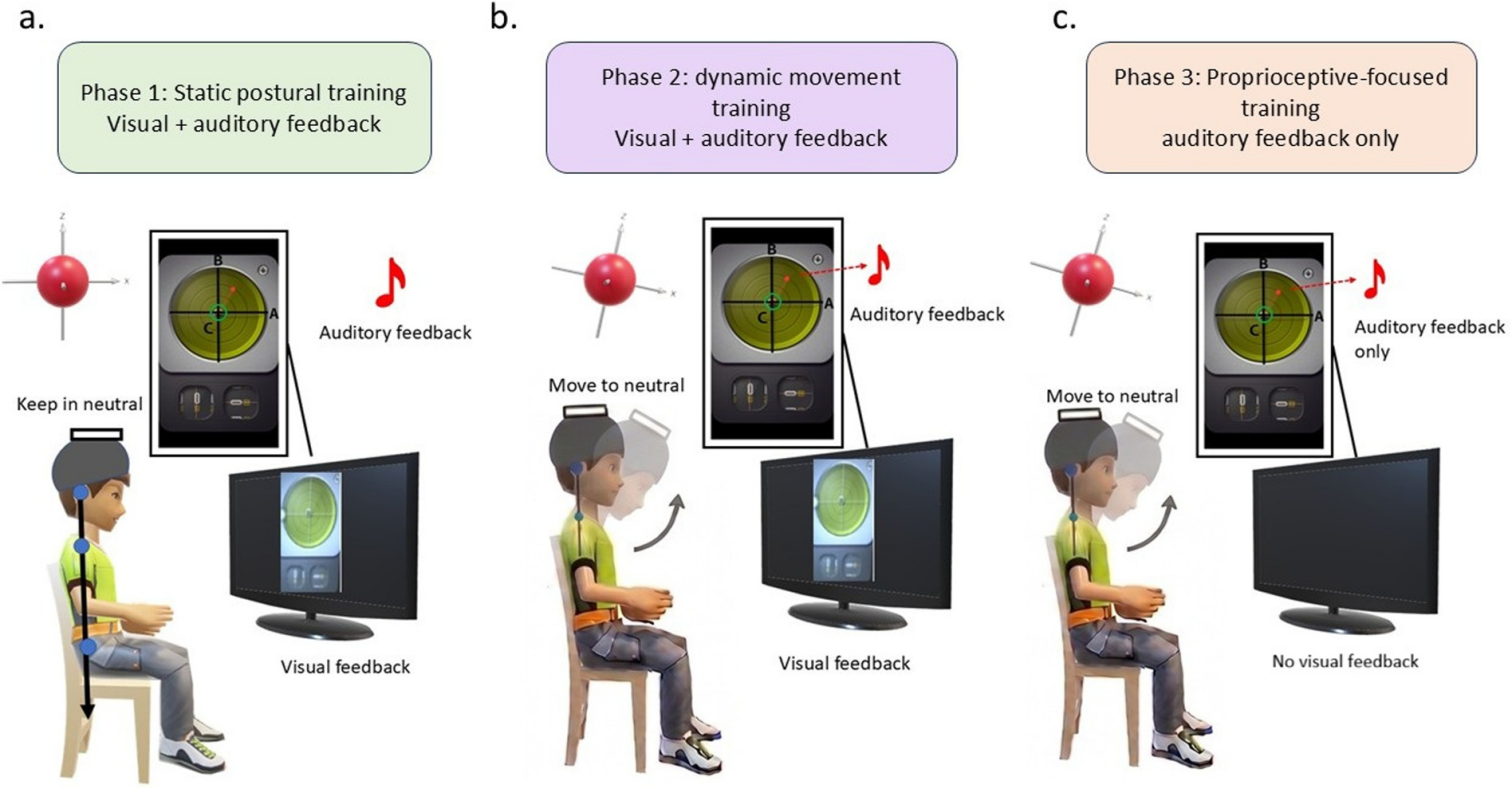
Sensorimotor biofeedback training protocol. The intervention progressed through three phases: (a) static postural training with visual and auditory feedback, (b) dynamic cervical movement training with visual and auditory feedback, and (c) proprioceptive‐focused training with reduced visual feedback. Visual feedback was displayed on the monitor as the target zone and current head position, whereas auditory feedback was triggered when the head position deviated outside the target boundary. A, B, and C indicate directional target positions, the green zone represents the acceptable target range, the cursor represents the participant's current head position, and the curved arrow indicates the required direction of cervical movement.

This study was a pilot randomized, parallel‐group trial designed to evaluate the short‐term effects of dynamic neck sensorimotor biofeedback training in individuals with mechanical neck pain. Participants were randomly allocated to either a training group or a control group using a lottery‐based randomization procedure. Group allocation was performed by an independent assistant who was not involved in participant recruitment, intervention delivery, or outcome assessment. Outcome measures were collected at baseline and after 2 weeks of intervention, as shown in Figure 1. Due to the nature of the intervention, participant blinding was not possible; outcome assessment was not blinded.

### 2.2 Subjects

A total of 20 participants with mechanical neck pain were recruited from the rehabilitation department of Kaohsiung Medical University Hospital. The diagnosis of mechanical neck pain was confirmed by a physician based on clinical examination. Inclusion criteria were: (1) neck pain attributed to musculoskeletal dysfunction; (2) pain localized between the occiput and scapular region; (3) myofascial neck pain; and (4) radiating head or neck pain without neurological deficits. Exclusion criteria included: (1) neck pain associated with neurological, rheumatic, or other pathological conditions; (2) a history of traumatic injury to the head or neck; (3) previous cervical spine surgery; (4) whiplash injury within the preceding 6 weeks; (5) pain originating from non‐cervical sources; and (6) neurological symptoms affecting the upper limbs.

Given the pilot nature of the study, the sample size was determined by feasibility within the study period rather than by formal power calculation. All participants received a detailed explanation of the study procedures and provided written informed consent prior to participation. The study procedures and consent forms were approved by the institutional review board of Kaohsiung Medical University Hospital (KMUH‐IRB‐20130102), and the trial has been registered on the ISRCTN registry no. ISRCTN52701452.

### 2.3 Procedure

#### 2.3.1 Training Group

Participants assigned to the training group received supervised sensorimotor biofeedback training in a seated position administered by a licensed physical therapist. Cervical motion and posture were monitored using a helmet‐mounted smartphone equipped with a motion‐sensing application that provided real‐time visual and auditory feedback displayed on an external monitor. Participants were instructed to adopt a relaxed upright posture, with the earlobe, acromion, and greater trochanter aligned vertically, to standardize the initial cervical and trunk positions. A 10‐min familiarization session was provided prior to the formal intervention to ensure task understanding and equipment adaptation.

The intervention was delivered twice weekly over a 2‐week period and consisted of three progressively structured phases designed to facilitate sensorimotor retraining through graded feedback. Each training session lasted approximately 60 min and included three 15‐min phases separated by 5‐min rest periods. For each phase, cervical movements in six directions (flexion, extension, left and right lateral flexion, and left and right rotation) were performed, with each movement repeated 20 times per set.

**FIGURE 2:**
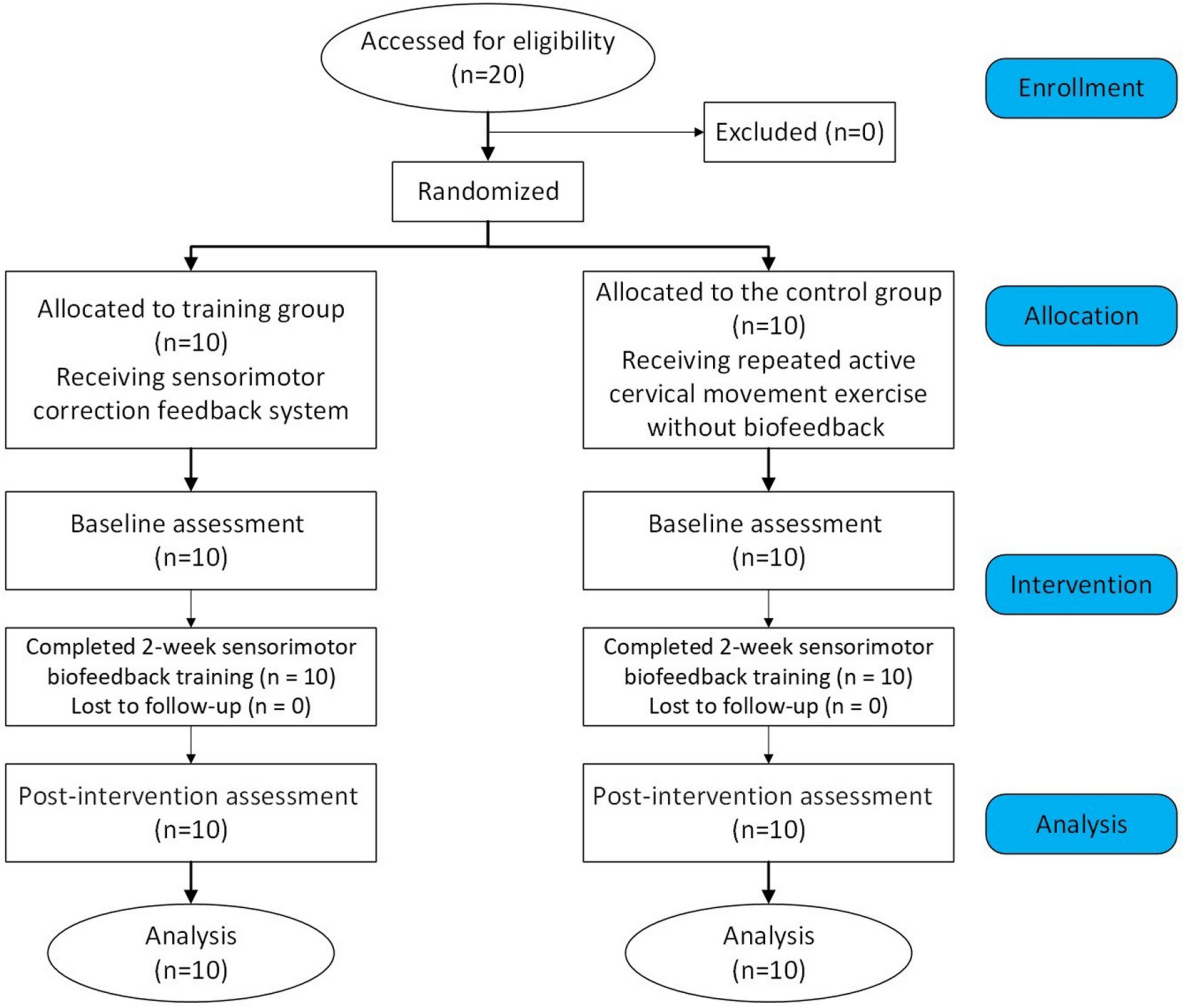
CONSORT participant flow diagram.

In Phase 1 (static postural training with multimodal feedback), participants were required to maintain a neutral cervical posture while seated. Visual feedback was provided by maintaining a cursor within a predefined target zone on the monitor, and auditory feedback was triggered when postural deviation exceeded the target boundary. Phase 2 (dynamic movement training with multimodal feedback) involved active cervical movements across the six directions, with concurrent visual and auditory feedback guiding movement accuracy and trajectory control in real time. In Phase 3 (proprioceptive‐focused training with reduced external feedback), participants repeated the same cervical movements with their eyes closed, relying primarily on auditory feedback to reinforce internal proprioceptive cues and promote sensorimotor integration. A schematic overview of the training setup and protocol is presented in Figure 2.

#### 2.3.2 Control Group

Participants in the control group performed the same sequence of cervical movements as the training group (static neutral holding, dynamic flexion–extension, side‐bending, and rotation, each repeated 20 times per direction), with identical session duration and frequency. However, no visual or auditory feedback was provided during the exercises. This design ensured that both groups received equal motor practice and cervical movements, isolating the effect of multimodal feedback on proprioception and clinical outcomes.

### 2.4 Outcome Measurements

Outcome measures were selected to reflect the sensorimotor focus of the intervention and its potential clinical relevance. Repositioning error (RPE), reflecting cervical proprioceptive acuity, was specified as the primary outcome because it most directly corresponded to the intended training target of head relocation accuracy and sensorimotor control. Secondary outcomes included other kinematic measures, namely maximal cervical range of motion (ROM) and movement units (MU), as well as patient‐reported clinical outcomes, including pain intensity, neck‐related disability, and fear‐avoidance beliefs.

#### 2.4.1 Kinematic Outcome Measurements

##### 2.4.1.1 Kinematic Data of the Cervical Joint

A six‐degree‐of‐freedom electromagnetic tracking system (Liberty, Polhemus Inc., USA) was used to assess head repositioning error, cervical mobility, and movement units in this study. This non‐invasive device determines sensor position by measuring the receiver's position relative to the transmitter, providing time‐series kinematic data. The system has been validated for reliability and accuracy, achieving a precision of ± 0.2° (Yang et al. 2012). The electromagnetic tracking system measured cervical movement in terms of position and angle with a sampling rate of 120 Hz. The system included three sensors: one on the forehead, one on the sternum (midpoint between jugular notch and xiphoid process), and a stylus receiver for referencing.

Cervical movements, including flexion, extension, bilateral rotation, and side‐bending, were randomly selected for testing; each movement was performed three times, and the maximum ROM value was recorded for analysis. MATLAB (R2007b, MathWorks, USA) was used to process time‐series data and calculate angular positions using Euler angle methods. Kinematic parameters included maximal ROM, angular velocity, and peak angular velocity across the tested planes.

##### 2.4.1.2 Head Repositioning Error (RPE)

Repositioning error (RPE) was assessed as the absolute value of the difference between the initial and final head positions. The initial position was defined as the first data point where angular velocity exceeded 5% of the peak, while the final position was the first data point where angular velocity fell below 5% of the peak and remained at that level for at least 0.5 s (Roijezon et al. 2008). Lower RPE values indicated better proprioceptive acuity. Each movement direction was tested three times, and the average was used for analysis.

##### 2.4.1.3 Movement Units (MUs)

Movement units (MUs) were used to quantify the smoothness of motion. An MU was defined as a continuous movement segment without a significant change in acceleration, with subsegments identified when the peak angular velocity exceeded 15% of the maximum (Kamper et al. 2002). A greater number of MUs indicated reduced movement smoothness. Cervical movements were randomly selected for testing; each movement was performed three times, and the average value was recorded for analysis.

#### 2.4.2 Subjective Outcome Measurements

Pain intensity was assessed using a 10‐point visual analog scale (VAS) (Ritter et al. 2006). Neck‐related disability was assessed using the Neck Disability Index (NDI; 10 items, total score 0–50, expressed as a percentage) (Vernon 2008). Fear‐avoidance beliefs were assessed using the Fear‐Avoidance Beliefs Questionnaire (FABQ), analyzed as FABQ‐PA (physical activity) and FABQ‐W (work) subscales (Waddell et al. 1993). The FABQ has demonstrated predictive validity in identifying patients at risk of prolonged disability and poor treatment outcomes (Cleland et al. 2008; Fritz and George 2002). This study analyzed FABQ‐PA and FABQ‐W separately to evaluate changes in fear‐related attitudes following intervention.

#### 2.4.3 Feasibility and Safety Outcomes

Feasibility outcomes included retention, adherence, and completeness of outcome data. Adherence was defined as completion of all four scheduled training sessions over the 2‐week intervention period. Safety was monitored by recording adverse events reported during training or assessment.

#### 2.4.4 Statistical Analysis

Statistical analyses were performed using SPSS software (version 20.0; SPSS Inc., Chicago, USA). Descriptive statistics (mean ± standard deviation, SD) were used to summarize participant characteristics, kinematic outcomes, and subjective measures. Data normality was assessed using the Shapiro–Wilk test. Baseline comparability between groups was evaluated using descriptive statistics and standardized mean differences (SMDs), with values < 0.2 considered negligible, 0.2–0.5 small, 0.5–0.8 moderate, and > 0.8 large.

Given the pilot nature of the trial, analyses emphasized the estimation of intervention‐related changes rather than definitive hypothesis testing. Within‐group change scores (post–pre) were summarized for each outcome, and within‐group pre–post comparisons were examined using paired tests when appropriate (paired *t*‐tests for approximately normal data or Wilcoxon signed‐rank tests otherwise). Between‐group effects were summarized as differences in mean change scores (training minus control) with uncertainty estimates (standard errors and 95% confidence intervals). Effect sizes were calculated using Cohen's *d*. For correlation analyses, the percentage change was calculated as (post − pre)/pre × 100%. Exploratory associations between changes in kinematic outcomes and patient‐reported outcomes were examined using Spearman's rank correlation coefficients. Exploratory multiple regression analyses were further used to investigate potential predictors of changes in patient‐reported outcomes. Given the pilot nature of the study and small sample size, these analyses were considered exploratory and hypothesis‐generating rather than confirmatory. Statistical significance was set at *p* < 0.05.

## 3 Results

### 3.1 Participant Flow, Feasibility, and Baseline Characteristics

A total of 20 participants were randomized to the training group (*n* = 10) or control group (*n* = 10), and all completed the intervention and post‐intervention assessments with no missing data (Figure 2). Feasibility outcomes were favorable: adherence in the training group was 100% (10/10 participants completed all four scheduled sessions), and no adverse events were reported. Baseline demographic, clinical, and kinematic characteristics were generally comparable between groups (Table 1). Standardized mean differences (SMDs) were mostly negligible to moderate imbalances, with moderate differences observed for pain intensity (VAS, SMD = 0.55), neck‐related disability (NDI, SMD = 0.47), cervical flexion ROM (SMD = 0.65), and right‐rotation repositioning error (RPE, SMD = −0.61).

**TABLE 1:** Characteristics of the training group and control group at baseline.

Variable (unit)	Movement	Training group	Control group	*p*‐value^a^	SMD
		Mean (SD)	Mean (SD)		
Age (year)		42.40 (16.77)	37.30 (14.68)	0.71	0.30
Height (cm)		160.80 (8.85)	161.30 (5.64)	0.85	−0.07
Weight (kg)		63.80 (16.32)	57.60 (12.28)	0.50	0.42
Onset duration	(Months)	5.68 (2.64)	6.32 (2.89)	0.79	−0.23
VAS		5.80 (2.10)	4.70 (1.83)	0.45	0.55
NDI		8.50 (3.75)	6.60 (4.12)	0.84	0.47
FABQ	Physical activity	13.30 (7.40)	13.50 (6.11)	1.00	−0.03
	Work	25.50 (10.66)	28.40 (9.17)	0.47	−0.29
ROM (degree)	F	55.73 (11.01)	48.55 (11.08)	0.16	0.65
	E	48.51 (10.93)	44.53 (11.34)	0.43	0.35
	R‐R	57.14 (6.33)	54.34 (9.72)	0.46	0.32
	R‐L	55.90 (8.36)	53.04 (11.32)	0.53	0.28
	S‐R	34.51 (6.61)	33.61 (8.26)	0.79	0.12
	S‐L	32.81 (6.80)	32.47 (7.32)	0.92	0.05
RPE (degree)		7.95 (4.80)	6.15 (2.24)	0.30	0.47
	E	7.33 (3.49)	6.65 (3.44)	0.67	0.20
	R‐R	3.94 (2.55)	6.05 (4.15)	0.19	−0.61
	R‐L	4.63 (3.29)	4.88 (2.36)	0.85	−0.08
	S‐R	3.29 (1.67)	3.31 (2.12)	0.98	−0.01
	S‐L	4.91 (1.26)	4.07 (2.79)	0.40	0.39
MU	F	5.22 (3.17)	5.55 (3.57)	0.83	−0.10
	E	5.13 (2.86)	5.97 (6.29)	0.71	−0.17
	R‐R	3.70 (2.32)	5.08 (4.75)	0.42	−0.38
	R‐L	4.87 (3.35)	4.85 (4.40)	0.99	< 0.01
	S‐R	5.60 (5.02)	4.68 (3.65)	0.65	0.20
	S‐L	5.57 (4.47)	4.83 (3.96)	0.70	0.17

Abbreviations: E, extension; F, flexion; FABQ, fear‐avoidance beliefs questionnaire; MU, movement units; NDI, neck disability index; R‐R/L, rotation to right/left; ROM, range of motion; RPE, repositioning absolute error; S‐R/L, side‐bending to right/left; SD, standard deviation; SMD, standardized mean difference; VAS, visual analog scale.

^a^ *p*‐value was calculated using the Wilcoxon rank sum test. Statistical significance was set at *p* < 0.05.

### 3.2 Kinematic Measurements

As shown in Table 2, the training group demonstrated reductions in repositioning error (RPE) following the 2‐week intervention. RPE decreased in flexion (7.95° ± 4.80 vs. 3.82° ± 3.64, *p* = 0.04, *d* = −0.97), extension (7.33° ± 3.49 vs. 4.05° ± 2.78, *p* = 0.02, *d* = −1.04). A significant reduction was also observed in right rotation (3.90° ± 2.55 vs. 2.55° ± 2.40, *p* = 0.047, *d* = −0.56), and left side bending (4.91° ± 1.26 vs. 3.21° ± 2.31, *p* = 0.02, *d* = −0.91). In the control group, RPE changes were generally small and did not reach statistical significance across planes.

**TABLE 2:** Comparison of pre‐ and post‐intervention changes in kinematic and subjective measures between the training and control groups.

Variable (unit)	Position	Training group, mean (SD)				Control group, mean (SD)			
		Pre	Post	*p* value	Cohen's *d*	Pre	Post	*p* value	Cohen's *d*
VAS		5.80 (2.10)	4.00 (1.63)	0.10	−0.96	4.70 (1.83)	4.55 (1.61)	0.45	−0.09
NDI		8.50 (3.75)	5.00 (2.79)	0.06	−1.06	6.60 (4.12)	6.00 (3.16)	0.84	−0.16
FABQ	PA	13.30 (7.40)	8.80 (6.75)	0.03^a^	−0.64	13.50 (6.11)	13.00 (6.98)	0.40	−0.08
	W	25.50 (10.66)	17.20 (9.84)	0.03^a^	−0.81	28.40 (9.17)	25.00 (9.76)	0.26	−0.36
ROM (degree)	F	55.73 (11.01)	60.33 (11.02)	0.06	0.42	48.55 (11.08)	48.67 (13.27)	0.96	0.01
	E	48.51 (10.93)	48.57 (8.39)	0.72	0.01	44.53 (11.34)	44.94 (11.14)	0.80	0.04
	R‐R	57.14 (6.33)	54.79 (8.83)	0.65	−0.31	54.34 (9.72)	50.79 (11.77)	0.04^a^	−0.33
	R‐L	55.90 (8.36)	57.29 (7.40)	0.45	0.17	53.04 (11.32)	47.26 (9.71)	0.20	−0.54
	S‐R	34.51 (6.61)	36.31 (12.37)	0.96	0.17	33.61 (8.26)	34.29 (8.59)	0.72	0.08
	S‐L	32.81 (6.80)	33.09 (6.07)	0.39	0.04	32.47 (7.32)	30.98 (8.41)	0.23	−0.19
RPE (degree)	F	7.95 (4.80)	3.82 (3.64)	0.04^a^	−0.97	6.15 (2.24)	5.57 (2.18)	0.06	−0.26
	E	7.33 (3.49)	4.05 (2.78)	0.02^a^	−1.04	6.65 (3.44)	6.36 (4.46)	0.45	−0.07
	R‐R	3.94 (2.55)	2.55 (2.40)	0.047^a^	−0.56	6.05 (4.15)	5.97 (4.58)	1.00	−0.02
	R‐L	4.63 (3.29)	3.21 (2.30)	0.14	−0.50	4.88 (2.36)	5.58 (3.58)	0.20	0.22
	S‐R	3.29 (1.67)	1.97 (1.24)	0.06	−0.89	3.31 (2.12)	4.03 (3.16)	0.96	0.26
	S‐L	4.91 (1.26)	3.21 (2.31)	0.02^a^	−0.91	4.07 (2.79)	4.71 (3.38)	0.26	0.21
MU	F	5.22 (3.17)	5.27 (2.62)	0.68	0.02	5.55 (3.57)	4.13 (2.45)	0.12	−0.46
	E	5.13 (2.86)	5.15 (2.31)	0.96	0.01	5.97 (6.29)	4.45 (4.11)	0.04^a^	−0.28
	R‐R	3.70 (2.32)	2.90 (0.99)	0.59	−0.43	5.08 (4.75)	3.60 (2.42)	0.01^a^	−0.39
	R‐L	4.87 (3.35)	2.98 (1.21)	0.07	−0.69	4.85 (4.40)	3.88 (3.11)	0.05	−0.24
	S‐R	5.60 (5.02)	4.03 (1.97)	0.17	−0.42	4.68 (3.65)	3.80 (2.34)	0.48	−0.27
	S‐L	5.57 (4.47)	3.75 (1.31)	0.33	−0.51	4.83 (3.96)	4.23 (2.82)	0.31	−0.17

Abbreviations: E, extension; F, flexion; FABQ, fear‐avoidance beliefs questionnaire; MU, movement units; NDI, neck disability index; PA, physical activity; R‐R/L, rotation to right/left; ROM, range of motion; RPE, repositioning absolute error; S‐R/L, side‐bending to right/left; SD, standard deviation; VAS, visual analog scale; W, work.

^a^ Level of significance using the Wilcoxon rank test.

Maximal cervical ROM was largely unchanged across most planes in both groups. In the training group, flexion ROM increased from 55.73° ± 11.01° to 60.33° ± 11.02 (*p* = 0.06, *d* = 0.42), whereas most other ROM directions remained broadly stable. In the control group, a reduction in right‐rotation ROM was observed (54.34° ± 9.72° vs. 50.79° ± 11.77°, *p* = 0.037, *d* = −0.33). Movement smoothness, quantified as movement units (MUs), also showed mixed changes across planes in both groups (Table 2).

### 3.3 Subjective Measurements

Within the training group, all subjective measures improved following the intervention (Table 2). Reductions in pain intensity (VAS) and neck‐related disability (NDI) did not reach statistical significance; however, both demonstrated large within‐group effect sizes (VAS: *d* = −0.96; NDI: *d* = −1.06). In contrast, fear‐avoidance beliefs demonstrated statistically significant within‐group improvements in the training group. FABQ‐PA declined from 13.30 ± 7.40 to 8.80 ± 6.75 (*p* = 0.03; *d* = −0.64), and FABQ‐W decreased from 25.50 ± 10.66 to 17.20 ± 9.84 (*p* = 0.03; *d* = −0.81). Changes in the control group were smaller in magnitude across these measures. As supportive comparisons, differences in change scores between groups generally favored the training group for proprioceptive acuity and fear‐avoidance beliefs, whereas changes in ROM and MU were mixed across planes (Table 2).

### 3.4 Associations Between Kinematic Improvements and Perceived Symptom Changes

Exploratory analyses within the training group suggested associations between percentage changes in proprioceptive performance and symptom‐related outcomes (Table 3). Pain intensity was positively correlated with RPE (*r* = 0.76, *p* < 0.05) and with NDI (*r* = 0.66, *p* < 0.05), indicating that larger proprioceptive errors were associated with greater pain and disability. FABQ‐PA was negatively correlated with maximal ROM (*r* = −0.71, *p* < 0.05), suggesting that greater cervical mobility was associated with lower fear‐avoidance beliefs regarding physical activity. No significant correlations were observed between MU and other variables.

**TABLE 3:** Spearman's rank correlation analysis of percentage changes in outcome variables.

	Pain	NDI	FABQ PA	FABQ W	RPE	MU	MAX ROM
Pain	1						
NDI	0.66^a^	1					
FABQ PA	0.05	0.02	1				
FABQ W	0.35	0.66^a^	0.27	1			
RPE	0.76^a^	0.50	−0.10	0.18	1		
MU	−0.36	0.10	0.01	0.15	−0.59	1	
MAX ROM	0.38	0.31	−0.71^a^	0.20	0.53	−0.26	1

Abbreviations: FABQ PA, fear‐avoidance beliefs questionnaire in physical activity; FABQ W, fear‐avoidance beliefs questionnaire in work; MAX ROM, maximal range of motion; MU, movement units; NDI, neck disability index; RPE, repositioning absolute error.

^a^ Level of significance using the Spearman rank correlation analysis. Percentage change = (post – pre)/pre × 100%.

In exploratory regression analyses, reductions in RPE were associated with VAS change in this sample (*R* ^2^ = 0.68, *p* = 0.003), while increases in ROM were associated with FABQ‐PA change (*R* ^2^ = 0.46, *p* = 0.031). No significant predictors were identified for changes in NDI or FABQ‐W. Given the pilot sample size, these association findings should be interpreted cautiously and considered hypothesis‐generating.

## 4 Discussion

This pilot randomized trial compared dynamic cervical sensorimotor training incorporating visual and auditory biofeedback with repeated cervical movement exercises without feedback in individuals with mechanical neck pain. The findings showed short‐term preliminary signals suggesting a potential added value of multimodal feedback, particularly for cervical proprioceptive acuity and fear‐avoidance beliefs. The most consistent changes were observed for RPE, particularly during flexion and extension, and for FABQ‐PA/FABQ‐W. In comparison, ROM and MU changes differed by movement direction and were less consistent, suggesting that these outcomes may be influenced by task characteristics and repeated exposure. Pain intensity and NDI showed favorable numerical reductions with large within‐group effect sizes in the training group; however, these changes did not reach statistical significance, and their clinical importance remains uncertain. Because small‐to‐moderate baseline differences were observed in some clinical and kinematic variables, the observed changes may have been influenced by baseline severity and should be interpreted cautiously, particularly for direction‐specific kinematic outcomes. Exploratory association analyses further indicated relationships between proprioceptive improvement and symptom change, but these findings should be considered hypothesis‐generating because of the small sample size.

The present findings are consistent with prior research showing that individuals with MNP commonly demonstrate impaired cervical proprioception, particularly during head repositioning tasks. Falla et al. (2004) reported increased RPEs in individuals with chronic neck pain, particularly during flexion–extension movements (Falla et al. 2004). Consistent with this literature, the reductions in RPE seen in our training group ‐especially during flexion and extension‐ are compatible with the possibility that feedback‐focused practice might facilitate sensorimotor recalibration by enhancing afferent input and movement control. This interpretation is also consistent with review‐level evidence framing neck pain as involving impaired cervical proprioception and sensorimotor control, supporting the rationale for proprioceptive and muscle‐coordination retraining in MNP (Zaidi et al. 2025). A plausible explanation relates to the intervention's progressive structure, which combined static postural stabilization with dynamic movement practice while gradually reducing external cues. This method is consistent with motor learning principles that focus on task progression and faded feedback to encourage error‐based learning and greater reliance on internal sensory cues (G. A. Jull et al. 2009; Winstein and Schmidt 1990). In the present study, the training tasks specifically required participants to control cervical posture and return the head toward a target position, which may have directly challenged the relocation ability reflected by RPE. Prior evidence also indicates that cervical proprioceptive training and craniocervical flexion training can improve cervical joint position sense, possibly through enhanced cervical afferent input or direct training of the sense of relocation (G. Jull et al. 2007; G. A. Jull et al. 2009). Although neuromuscular mechanisms were not directly assessed in the present study, craniocervical flexion training has been shown to modify the spatial and temporal characteristics of deep cervical flexor activation (G. A. Jull et al. 2009), and altered cervical muscle activation patterns have been associated with disability in individuals with MNP (Bonilla‐Barba et al. 2020). These findings provide indirect support for a possible mechanism in which sensorimotor‐oriented training may influence pain‐related outcomes by improving cervical afferent input, deep cervical flexor control, movement confidence, and perceived safety during movement, rather than solely by correcting repositioning error. However, the relationship between proprioceptive improvement and pain reduction should be interpreted cautiously as current evidence supports a plausible association rather than a confirmed causal pathway.

The patient‐reported outcomes showed a mixed but clinically informative pattern. Pain intensity and NDI demonstrated favorable numerical reductions with large within‐group effect sizes in the training group, although neither change reached statistical significance. When compared with previously reported minimal clinically important change (MCIC) values, the 3.5‐point reduction in NDI was comparable to the MCIC reported by Pool et al., whereas the 1.8‐point reduction in pain intensity was close to but below the reported cutoff for clinically important pain improvement (Pool et al. 2007). These findings should therefore be viewed as favorable preliminary signals rather than as confirmed clinically meaningful effects. In contrast, FABQ‐PA and FABQ‐W showed statistically significant within‐group reductions, suggesting that fear‐avoidance beliefs may be particularly responsive to short‐term feedback‐guided sensorimotor training. This pattern aligns with the fear‐avoidance model and previous evidence indicating that pain‐related fear and movement avoidance contribute to disability and may be addressed through rehabilitation (Zale and Ditre 2015). Accordingly, the observed FABQ reductions may reflect a favorable preliminary change in pain‐related movement beliefs, although longer follow‐up is needed to determine whether these changes translate into sustained improvements in pain or disability.

The exploratory association between increased cervical flexion ROM and reduced FABQ‐PA may indicate a link between improved movement capacity and lower fear‐avoidance regarding physical activity. Batool et al. (2024) similarly reported that exercise interventions increasing ROM were accompanied by reductions in FABQ scores after 3 weeks of neck training (Batool et al. 2024). While ROM changes in the present study were not consistent across planes, sagittal‐plane mobility remains functionally relevant because individuals with chronic neck pain often show notable restrictions in flexion–extension (Falla et al. 2004). Previous work has further highlighted that flexion–extension movements occur nearly twice as often as movements in other planes, underlining their functional relevance in daily activities (Sterling et al. 2008). However, because cervical flexion ROM showed a moderate baseline difference between the groups, this direction‐specific finding should be interpreted cautiously. Together, these observations support the hypothesis that improving sagittal‐plane mobility may be related to breaking the cycle in which restricted movement reinforces fear and avoidance, consistent with evidence linking higher fear‐avoidance beliefs to greater long‐term disability risk (Lindstroem et al. 2012). Notably, the control group performing repeated cervical motion exercises also exhibited some plane‐specific ROM changes without corresponding decreases in fear‐avoidance beliefs, indicating that improvements in movement capacity may not automatically translate into reduced fear‐avoidance unless fear‐related movement beliefs are explicitly addressed as intervention targets.

Both groups exhibited MU changes, but interpretation requires caution. In the control group, MU reductions were accompanied by limited proprioceptive change, raising the possibility that smoother execution partly reflected test familiarity rather than substantive sensorimotor adaptation (O'Leary et al. 2012). Pain‐related protective responses may also constrain movement strategies and joint excursion (Batool et al. 2024). In the training group, MU changes occurred alongside more consistent proprioceptive improvements and, in some planes, improved ROM, which may reflect greater sensorimotor recalibration. However, because MU changes were inconsistent across planes and were also observed in the control group for certain directions, MU should be considered a supplementary, exploratory indicator in this pilot trial rather than a primary marker of intervention benefit.

This pilot trial contributes to neck pain rehabilitation research by evaluating a sensorimotor training approach that integrates task‐specific control with real‐time multisensory feedback and graded cue reduction. Importantly, the feasibility outcomes were favorable: retention and outcome completeness were high, adherence to the training protocol was 100%, and no adverse events were reported. These findings support the feasibility and short‐term safety of the protocol under supervised conditions. Together with the preliminary effect estimates, especially for RPE and FABQ outcomes, these feasibility findings can inform the selection of primary outcomes and the design of a larger, definitive randomized trial—for example, by determining the intervention duration, follow‐up schedule, and predefined analytical models. Future work should prioritize blinded outcome assessment where possible and confirm between‐group effects using adequately powered samples and prespecified statistical approaches.

### 4.1 Study Limitations

Several limitations should be acknowledged. First, as a pilot trial with a small sample size, the findings have limited generalizability and were not intended to provide definitive hypothesis testing; results should be interpreted as preliminary. Second, the intervention consisted of only four supervised sessions over 2 weeks, and the absence of follow‐up precludes conclusions regarding the durability of the observed changes. Third, outcome assessment was not blinded, which may introduce measurement bias although objective kinematic outcomes were included. Fourth, the number of statistical comparisons should be considered when interpreting the findings, particularly the exploratory correlation and regression analyses, which may be unstable in small samples and require confirmation with prespecified models in larger datasets. Finally, the lack of neurophysiological or electromyographic measures limits mechanistic interpretation, and the requirement for supervised biofeedback equipment may limit immediate translation to routine clinical practice. Future research with larger samples, longer follow‐up, blinded assessment, and mechanistic outcomes is warranted.

This pilot randomized trial provides preliminary evidence that a short‐term sensorimotor training program combining static and dynamic cervical tasks with progressively reduced biofeedback may improve cervical proprioceptive acuity and reduce fear‐avoidance beliefs in individuals with mechanical neck pain. Changes in range of motion and movement smoothness varied across movement directions and should be interpreted with caution. These findings suggest that proprioception‐focused, feedback‐guided sensorimotor retraining may be a promising adjunctive approach to exercise‐based rehabilitation for MNP, addressing proprioceptive deficits and fear‐avoidance, while larger trials determine the magnitude and durability of benefit. Future adequately powered studies with longer follow‐up and blinded assessment are warranted to confirm effectiveness, clarify mechanisms, and optimize training parameters.

## Author Contributions

**Min Hsu:** conceptualization, methodology, formal analysis, investigation, writing – original draft, writing – review and editing. **Yen‐Cheng Lee:** conceptualization, methodology, formal analysis, investigation, writing – original draft. **Chia‐Chi Yang:** methodology, formal analysis, writing – original draft. **Pei‐Chi Lin:** conceptualization, writing – original draft. **Lih‐Jiun Liaw:** investigation, writing – review and editing. **Chen‐Yu Chien:** investigation, writing – review and editing. **Pei‐Yun Lee:** writing – original draft, writing – review and editing. **Lan‐Yuen Guo:** conceptualization, methodology, formal analysis, investigation, writing – original draft, writing – review and editing, funding acquisition.

## Funding

This study was supported from the National Health Research Institutes (Grant NHRI‐EX114‐11227EI), the Precision Sports Medicine and Health Promotion Center, Kaohsiung Medical University, Kaohsiung 807, Taiwan (Grant KMU‐TC115A06‐1), the Research Center for Precision Environmental Medicine, Kaohsiung Medical University, Kaohsiung, Taiwan from The Featured Areas Research Center Program within the framework of the Higher Education Sprout Project by the Ministry of Education (MOE) in Taiwan and by Kaohsiung Medical University Research Center (Grant KMU‐TC115A01), and Ministry of Science and Technology, Taiwan (Grant NSTC114‐2622‐8‐037‐003).

## Ethics Statement

All study procedures were conducted in accordance with the principles outlined in the Declaration of Helsinki. The study's ethical approval was obtained from the Institutional Review Board of Kaohsiung Medical University Hospital (KMUH‐IRB‐20130102), and it has been registered on the ISRCTN registry (no. ISRCTN52701452).

## Consent

An informed consent form was signed by the patients prior to their participation in the study.

## Conflicts of Interest

The authors declare no conflicts of interest.

## Data Availability

Data supporting this study are available from the corresponding author upon reasonable request.
